# Supplementary material for: Factors That Influence the Sustainability of Human Milk Donation to Milk Banks: A Systematic Review
Source: Nutrients. 2022 Dec 9;14(24):5253. doi: 10.3390/nu14245253 (PMC9785923; doi:10.3390/nu14245253)
Supplement: Supplementary file 1 [file nutrients-14-05253-s001.zip › nutrients-2050934-supplementary.pdf]

Supplementary material

Supplementary Table S1 : PRISMA 2020 Checklist

Section and Topic	Item #	Checklist item	Location where item is reported
**TITLE**			
Title	1	Identify the report as a systematic review.	Page 1
**ABSTRACT**			
Abstract	2	See the PRISMA 2020 for Abstracts checklist.	Page # 1
**INTRODUCTION**			
Rationale	3	Describe the rationale for the review in the context of existing knowledge.	Page # 1-2
Objectives	4	Provide an explicit statement of the objective(s) or question(s) the review addresses.	Page # 2
**METHODS**			
Eligibility criteria	5	Specify the inclusion and exclusion criteria for the review and how studies were grouped for the syntheses.	Page # 3
Information sources	6	Specify all databases, registers, websites, organisations, reference lists and other sources searched or consulted to identify studies. Specify the date when each source was last searched or consulted.	Page # 2-3<br>Figure 1<br>Supplemental Table S2
Search strategy	7	Present the full search strategies for all databases, registers and websites, including any filters and limits used.	Page # 2-3<br>Supplemental Table S2
Selection process	8	Specify the methods used to decide whether a study met the inclusion criteria of the review, including how many reviewers screened each record and each report retrieved, whether they worked independently, and if applicable, details of automation tools used in the process.	Page # 3
Data collection process	9	Specify the methods used to collect data from reports, including how many reviewers collected data from each report, whether they worked independently, any processes for obtaining or confirming data from study investigators, and if applicable, details of automation tools used in the process.	Page # 3
Data items	10a	List and define all outcomes for which data were sought. Specify whether all results that were compatible with each outcome domain in each study were sought (e.g. for all measures, time points, analyses), and if not, the methods used to decide which results to collect.	Page # 3-4
	10b	List and define all other variables for which data were sought (e.g. participant and intervention characteristics, funding sources). Describe any assumptions made about any missing or unclear information.	Page # 3-4
Study risk of bias assessment	11	Specify the methods used to assess risk of bias in the included studies, including details of the tool(s) used, how many reviewers assessed each study and whether they worked independently, and if applicable, details of automation tools used in the process.	Page # 3-4
Effect measures	12	Specify for each outcome the effect measure(s) (e.g. risk ratio, mean difference) used in the synthesis or presentation of results.	N/A
Synthesis methods	13a	Describe the processes used to decide which studies were eligible for each synthesis (e.g. tabulating the study intervention characteristics and comparing	Page # 3-4

Section and Topic	Item #	Checklist item	Location where item is reported
		against the planned groups for each synthesis (item #5)).	
	13b	Describe any methods required to prepare the data for presentation or synthesis, such as handling of missing summary statistics, or data conversions.	N/A
	13c	Describe any methods used to tabulate or visually display results of individual studies and syntheses.	Page # 3-4& Table 2
	13d	Describe any methods used to synthesize results and provide a rationale for the choice(s). If meta-analysis was performed, describe the model(s), method(s) to identify the presence and extent of statistical heterogeneity, and software package(s) used.	Page # 3-4
	13e	Describe any methods used to explore possible causes of heterogeneity among study results (e.g. subgroup analysis, meta-regression).	N/A
	13f	Describe any sensitivity analyses conducted to assess robustness of the synthesized results.	N/A
Reporting bias assessment	14	Describe any methods used to assess risk of bias due to missing results in a synthesis (arising from reporting biases).	Page # 3-4 & Supplementary Table S3
Certainty assessment	15	Describe any methods used to assess certainty (or confidence) in the body of evidence for an outcome.	Page # 4 & Supplementary Table S4
**RESULTS**			
Study selection	16a	Describe the results of the search and selection process, from the number of records identified in the search to the number of studies included in the review, ideally using a flow diagram.	Page # 4 & Figure 1
	16b	Cite studies that might appear to meet the inclusion criteria, but which were excluded, and explain why they were excluded.	N/A
Study characteristics	17	Cite each included study and present its characteristics.	Page # 4-5 & Table 1
Risk of bias in studies	18	Present assessments of risk of bias for each included study.	Page # 6-8, Table 1 & details in Supplementary Table S3
Results of individual studies	19	For all outcomes, present, for each study: (a) summary statistics for each group (where appropriate) and (b) an effect estimate and its precision (e.g. confidence/credible interval), ideally using structured tables or plots.	Page # 5-14, Table 1 (& 2)
Results of syntheses	20a	For each synthesis, briefly summarise the characteristics and risk of bias among contributing studies.	Page # 5-14, Table 1, 2 & Supplementary Table S3
	20b	Present results of all statistical syntheses conducted. If meta-analysis was done, present for each the summary estimate and its precision (e.g. confidence/credible interval) and measures of statistical heterogeneity. If comparing groups, describe the direction of the effect.	N/A
	20c	Present results of all investigations of possible causes of heterogeneity among study results.	N/A

Section and Topic	Item #	Checklist item	Location where item is reported
	20d	Present results of all sensitivity analyses conducted to assess the robustness of the synthesized results.	N/A
Reporting biases	21	Present assessments of risk of bias due to missing results (arising from reporting biases) for each synthesis assessed.	N/A
Certainty of evidence	22	Present assessments of certainty (or confidence) in the body of evidence for each outcome assessed.	Page # 9-13 & Supplementary Table S4
**DISCUSSION**			
Discussion	23a	Provide a general interpretation of the results in the context of other evidence.	Page # 14-16
	23b	Discuss any limitations of the evidence included in the review.	Page # 16-17
	23c	Discuss any limitations of the review processes used.	Page # 16-17
	23d	Discuss implications of the results for practice, policy, and future research.	Page # 16-17
**OTHER INFORMATION**			
Registration and protocol	24a	Provide registration information for the review, including register name and registration number, or state that the review was not registered.	N/A
	24b	Indicate where the review protocol can be accessed, or state that a protocol was not prepared.	Page # 2
	24c	Describe and explain any amendments to information provided at registration or in the protocol.	Page # 2-3
Support	25	Describe sources of financial or non-financial support for the review, and the role of the funders or sponsors in the review.	Page # 17
Competing interests	26	Declare any competing interests of review authors.	Page # 17-18
Availability of data, code and other materials	27	Report which of the following are publicly available and where they can be found: template data collection forms; data extracted from included studies; data used for all analyses; analytic code; any other materials used in the review.	Supplementary Tables S3, S4

From: Page MJ, McKenzie JE, Bossuyt PM, Boutron I, Hoffmann TC, Mulrow CD, et al. The PRISMA 2020 statement: an updated guideline for reporting systematic reviews. BMJ 2021;372:n71. doi: 10.1136/bmj.n71

Supplementary Table S2a,b,c,d,e,f,g,h: search strategy

Table S2a: Medline (PubMed) database, last search on 30.01.2022:

Search steps	Search terms/descriptors	Number of hits
# 1	"Milk Banks"[Mesh] OR "milk bank*"[Title/Abstract] OR **"milkbank*"[Title/Abstract]**	1000
# 2	**"milk donation*"[Title/Abstract] OR "Donor human milk"[Title/Abstract] OR "Human Donor Milk"[Title/Abstract] OR "Human Milk Donor*"[Title/Abstract]**	513
# 3	#1 OR #2	1196

Table S2b: CINAHL, last search on 30.01.2022:

Search steps	Search terms/descriptors	Number of hits
# 1	(MH "Milk Banks") OR (TI “milk bank*”) OR (AB “milk bank*”)	751
# 2	TI milk donation* OR AB (milk donation*) OR (MH "Donor Milk") OR TI "human donor milk" OR AB "human donor milk" OR TI "donor human milk" OR AB "donor human milk" OR TI "human milk donor*" OR AB "human milk donor*"	471
# 3	#1 OR #2	962

Table S2c: EMBASE last search on 30.01.2022:

Search steps	Search terms/descriptors	Number of hits
# 1	'milk bank'/exp OR 'milk bank*':ti,ab,kw OR 'milkbank*':ti,ab,kw	990
# 2	'milk donation*':ti,ab,kw OR 'donor milk'/exp OR 'donor human milk':ti,ab,kw OR 'human donor milk':ti,ab,kw OR 'human milk donor*':ti,ab,kw	839
# 3	#1 OR #2	1490

Table S2d: Cochrane library, last search on 30.01.2022:

Search steps	Search term(s) /descriptors	Number of hits
# 1	(Milk Banks) OR (milk bank*) OR (milkbank*)	2
# 2	(milk donation*) OR (Donor human milk) OR (Human Donor Milk) OR (Human Milk Donor*)	11
# 3	1 OR 2	11

Table S2e: CENTRAL, last search on 30.01.2022:

Search steps	Search term(s) /descriptors	Number of hits
# 1	(Milk Banks) OR (milk bank*) OR (milkbank*)	76
# 2	(milk donation*) OR (Donor human milk) OR (Human Donor Milk) OR (Human Milk Donor*)	183
# 3	1 OR 2	227

Table S2f: PsycINFO, last search on 30.01.2022:

Search steps	Search term(s) /descriptors	Number of hits
# 1	(**Any Field**: "Milk Banks") *OR* (**Any Field**: "milk bank*") *OR* (**Any Field**: "milkbank*") *OR* (**Any Field**: "milk donation*") *OR* (**Any Field**: "milk of human donor*") *OR* (**Any Field**: "Donor human milk") *OR* (**Any Field**: "Human Donor Milk") *OR* (**Any Field**: "Human Milk Donor*")	40

Table S2g: Web of science, last search on 30.01.2022:

Search steps	Search term(s) /descriptors	Number of hits
# 1	("Milk Banks") OR ("milk bank*") OR ("milkbank*") OR ("milk donation*") OR ("milk of human donor*") OR ("Donor human milk") OR ("Human Donor Milk") OR ("Human Milk Donor*")	1367

Table S2h: SCOPUS, last search on 30.01.2022:

Search steps	Search term(s) /descriptors	Number of hits
# 1	TITLE-ABS-KEY ((*"milk bank*"* OR *"milk donation*"* OR *"Donor human milk"* OR *"Human Donor Milk"* OR *"Human Milk Donor*"*) AND *(EXCLUDE (PUBYEAR , 2022))*	1419

Supplementary Table S3a: Risk of bias of quantitative studies

	1. Was the research question or objective in this paper clearly stated?	2. Was the study population clearly specified and defined?	3. Was the participation rate of eligible persons at least 50%?	4. Were all the subjects selected or recruited from the same or similar populations (including the same time period)? Were inclusion and exclusion criteria for being in the study prespecified and applied uniformly to all participants?	5. Was a sample size justification, power description, or variance and effect estimates provided?	6. For the analyses in this paper, were the exposure(s) of interest measured prior to the outcome(s) being measured?	7. Was the timeframe sufficient so that one could reasonably expect to see an association between exposure and outcome if it existed?	8. For exposures that can vary in amount or level, did the study examine different levels of the exposure as related to the outcome (e.g., categories of exposure, or exposure measured as continuous variable)?	9. Were the exposure measures (independent variables) clearly defined, valid, reliable, and implemented consistently across all study participants?	10. Was the exposure(s) assessed more than once over time?	11. Were the outcome measures (dependent variables) clearly defined, valid, reliable, and implemented consistently across all study participants?	12. Were the outcome assessors blinded to the exposure status of participants?	13. Was loss to follow-up after baseline 20% or less?	14. Were key potential confounding variables measured and adjusted statistically for their impact on the relationship between exposure(s) and outcome(s)?	Quality rating
Alencar & Seidl, 2010	Yes<br>The aim is clearly stated.	Yes<br>Population clearly specified and defined.	No<br>The 36 study participants represented only 20.2% of the total 178 human milk banks donors.	Yes<br>All subjects were recruited from same population.	No<br>No description of sample size calculation or justification, no power description or variance and effect estimates provided in the article.	No<br>All variables were measured at the same time.	No<br>Observational descriptive study with no time to see an effect.	Not applicable<br>Not applicable.	Cannot determine<br>Were self-reported	Not applicable<br>Not applicable: cross sectional study where the exposures were assessed once.	Not applicable<br>Not applicable (no dependent variable reported)	Not applicable<br>not applicable	Not applicable<br>Not applicable (not a cohort study).	Not applicable.	High risk of bias. Descriptive crossectional study with a participation rate of only 20.2% of eligible persons. Very small sample size (n=36). No multivariate (regression analysis…) reported. Weak quality.
Bocci et al., 2019	Yes<br>Aim clearly described.	Yes<br>Population clearly specified and defined.	Yes<br>Census of all the eligible persons.	Yes<br>Census of all eligible person, no exclusion criteria reported.	No<br>No description of sample size calculation or justification, no power description or variance and effect estimates provided in the article.	No<br>All variables were measured at the same time.	No<br>Independant and dependant variables assessed at the same time.	Not applicable<br>Not applicable as the independent variables are dichotomous.	Cannot determine<br>Cannot determine as data was extracted from medical records but it is not clear how the independent	Not applicable<br>Not applicable (retrospect-ive observational study)	Cannot determine<br>The dependent variable was defined but the way it was measured is not specified in the article.	Not applicable<br>not applicable	Not applicable<br>Not applicable (not a cohort study).	Yes<br>The result of a multiple linear regression is reported in the article, however no information are displayed on the ref. category used.	Low risk of bias<br>The result of a multiple linear regression is reported in the article but no information are displayed on the reference category used. Good quality.

									variables were measured.						
Jarmoc et al., 2021	Yes<br>Aim clearly stated.	Yes<br>Population clearly specified and defined.	Yes<br>3764 of the 4181 human milk donors included in the original dataset of the Human milk bank were included in this study (approximately 90%).	Yes<br>All subjects recruited from same population. Inclusion and exclusion criteria seemed to be applied uniformly.	No<br>No description of sample size calculation or justification, no power description or variance and effect estimates provided in the article.	Yes<br>The exposure preceded the outcome	Yes<br>Timeframe (< 8 years) seems sufficient to see an association between independent variable and dependent variable if it existed.	Not applicable<br>Not applicable as the independent variables are dichotomous.	Cannot determine<br>Cannot determine as it is not clearly reported how the independent variables were measured.	Not reported<br>Not reported in the article.	Cannot determine<br>Cannot determine: The dependent variable was defined but the way it was measured is not specified in the article.	Not reported<br>Not reported in the article.	Not applicable<br>Not applicable	Yes<br>Regression analyses were performed.	Low risk of bias. Methodology evaluated as good.
Nangia et al., 2020	Yes<br>The objectives of the study were clearly stated.	Yes<br>The study population was clearly specified and defined.	Yes<br>All eligible persons were included (census).	Yes<br>All subjects were recruited from same population, and appear to have been selected based on the same inclusion criteria (no exclusion criteria were reported).	No<br>No description of sample size calculation or justification, no power description or variance and effect estimates provided in the article.	No<br>All variables were measured at the same time.	No<br>All variables were measured at the same time.	Yes<br>Most of the independent variables are categorical (but not dichotom-ous).	Cannot determine<br>All the independent variables were briefly described and seems to be objective however their is no information on measures' accuracy and reliability (for example: same scale, same measurer for all of the infants' weights?).	Not applicable<br>Not applicable: cross sectional study where the exposures were assessed once.	Cannot determine<br>Cannot determine because the dependent variable was defined but the way it was measured is not specified in the article.	Not applicable<br>not applicable	Not applicable<br>Not applicable (not a cohort study).	No<br>No report of potential confounding variables measured and adjusted for (no report of the use of any regression analysis).	Moderate risk of bias Moderate methodological quality: no report of potential confounding variables measured and adjusted for (no report of any regression analysis)
Osbaldist-on & Mingle, 2007	Yes<br>The objectives of the study were clearly stated.	Yes<br>The study population was clearly specified and defined.	No<br>Of the 324 donors contacted, only 35% responded n= 114, among whom 76% agreed to schedule an interview and participated in the study.	No<br>Study participants were not recruited among the same population and their inclusion criteria differed between the	No<br>No description of sample size calculation or justification, no power description or variance and effect estimates provided in the article.	No<br>All variables were measured at the same time.	No<br>All variables were measured at the same time.	Yes<br>Some independent variables that can vary in amount were categorical (but not dichotomous).	Cannot determine<br>Cannot determine as it is not clearly reported how the independent variables were measured.	Not applicable<br>Not applicable: cross sectional study where the independent variables were assessed once.	Yes<br>Objective measurement by the milk bank staff (not self-reported by donors.	Not applicable<br>not applicable	Not applicable<br>Not applicable (not a cohort study).	No<br>No report of potential confounding variables measured and adjusted for (no report of the use of any regression analysis).	Moderate risk of bias Moderate methodological quality: response rate <35%, participants self-selected. Small sample size. No report of potential confounding variables measured and

				donor and non-donor groups.											adjusted for (no report of any regression analysis).
Pimenteira Thomaz et al., 2008	Yes<br>The aim was clearly stated.	Yes<br>The study population was clearly specified and defined.	Cannot determine<br>Could not determine how much the participation rate of eligible persons was (what percentage of the target population does the 737 human milk donors represent)?	Yes<br>All subjects were recruited from same population.	No<br>No description of sample size calculation or justification, no power description or variance and effect estimates provided in the article.	No<br>All variables were measured at the same time.	No<br>All variables were measured at the same time.	Yes<br>Most of the independent variables that can vary in amount were categorical (but not dichotomous).	No<br>Answers were self-reported.	Not applicable<br>Not applicable: cross sectional study where the exposures were assessed once.	No<br>The dependent variable was self-reported.	Not applicable<br>Not applicable.	Not applicab le<br>Not applicab le (not a cohort study).	Yes<br>Regression analyses were performed.	Moderate risk of bias.<br>Independent and dependent variables were self-reported. Unclear how much the participation rate of eligible persons was (what percentage of the target population does the 737 human milk donors represent)...
Quitadamo et al., 2018	Yes<br>Aim clearly stated.	Yes<br>Population clearly specified and defined.	Not reported<br>The article does not mention what percentage of the target population the 90 donors represent.	Yes<br>All subjects recruited from same population.	No<br>No description of sample size calculation or justification, no power description or variance and effect estimates provided in the article.	No<br>All variables were measured at the same time.	No<br>Exposure and outcomes assessed at the same time.	Not applicable<br>Not applicable as the independent variables are dichotomous .	Cannot determine<br>Cannot determine as it is not clearly reported how each of the independent variables were measured. The logic behind the Job categories group 1 and group 2 is unclear.	Not applicable<br>Not applicable: cross sectional study where the exposures were assessed once.	Cannot determine<br>Cannot determine because the dependent variable was defined but the way it was measured is not specified in the article.	Not applicable<br>not applicable	Not applicab le<br>Not applicab le (not a cohort study).	No<br>No report of potential confounding variables measured and adjusted for (no report of any regression analysis)	High risk of bias<br>Weak methodology quality: small sample size. Could not determine what percentage of the target population the 90 donors represent.No report of potential confounding variables measured and adjusted for (no report of any regression analysis).

Sierra-Colomina et al., 2014	Yes<br>The aim is clearly stated.	Yes<br>Population clearly specified and defined.	Yes<br>Census of all the donors accepted in the human milk bank during the period of the study and who had already finished their donation.	Yes<br>All subjects recruited from same population.	No<br>No description of sample size calculation or justification, no power description or variance and effect estimates provided in the article.	No<br>All variables were measured at the same time.	No<br>All variables were measured at the same time.	Yes<br>Some independent variables that can vary in amount were categorical (but not dichotomous).	Cannot determine<br>Cannot determine. Most independent variable seems self reported.	Not applicable<br>Not applicable: cross sectional study where the independent variables were assessed once.	Cannot determine<br>Cannot determine because the dependent variable was defined but the way it was measured is not clear.	Not applicable<br>Not applicable.	Not applicable<br>Not applicable (not a cohort study).	Yes<br>Regression analyses were performed and explained.	Low risk of bias. Methodology evaluated as good.

Supplementary Table S3b : Risk of bias of qualitative studies

Observational cohort and cross-sectional studies	SECTION A: Are the results valid? 1. Was there a clear statement of the aims of the research?	2. Is a qualitative methodology appropriate?	3. Was the research design appropriate to address the aims of the research?	4. Was the recruitment strategy appropriate to the aims of the research?	5. Was the data collected in a way that addressed the research issue?	6. Has the relationship between researcher and participants been adequately considered?	SECTION B : What are the results? 7. Have ethical issues been taken into consideration?	8. Was the data analysis sufficiently rigorous?	Section C: Will the results help locally? 9. Is there a clear statement of findings?	10. How valuable is the research?	Quality rating
Machado et al., 2015	Yes<br>The aim was clearly stated as well as its relevance.	Yes<br>The methodology seems appropriate.	Yes<br>The research design seems appropriate to address the aim and was very briefly explained in the article.	Can't Tell<br>The article only mentions that it is a convenience sample, but participant selection (on which criteria they were selected…) is not described.	Can't Tell<br>Data collected through semi-structured interviews. The methods chosen were not justified and the form of data as well as data saturation were not mentioned or discussed.	Can't Tell<br>Not mentioned in the article.	Yes<br>Ethical approval was obtained from the ethics committee and consent was gathered from all the participants.	Yes<br>The analysis process was described and data were presented to support the findings.	Yes<br>The findings are explicit (but their credibility is not discussed).	Yes<br>The contribution this study makes is briefly mentioned but the transferability of the findings cannot be assumed due to the small convenience sample size.	**Good quality:** The methodology seems appropriate and the findings are explicitly stated, although transferability cannot be assumed due to the small convenience sample size. Unclear participant selection.
Mondkar et al., 2018	Yes<br>The aim was clearly stated.	Yes<br>A qualitative methodology is approriate to address the aim of this research.	Yes<br>The authors did not justify the research design in the article, but it seems appropriate.	Can't Tell<br>The authors described briefly how the participants were selected but did not discuss the recruitment process.	Yes<br>Data was collected in a way that addressed the research issue (setting was justified, data collection methods were explained, and data form was mentioned).	Can't Tell<br>The authors piloted the study but no other information was displayed in the article about whether the relationship between researcher and participants had been adequately considered.	Yes<br>Ethical approval was obtained from the ethics committee and consent was gathered from all the participants.	Can't Tell<br>The analysis process was described. However, the extent to which contradictory data were taken into account was not mentioned and the authors did not discuss critically their own role, potential bias and influence during analysis and selection of data for presentation.	Yes<br>The findings are explicit and are discussed in relation to the original research question.	Yes<br>The researchers discussed the contributions of this study and its importance in the Indian context.	**Good quality:**<br>the methodology seems appropriate and choices were justified. Unclear participants' recruitment process. Findings and their contributions were discussed, as well as their importance in the Indian context.

Supplementary Table S4a: GRADE Quantitative Evidence profile

	Summary review findings	Studies contributing to the review	Risk of Bias	Inconsistency	Indirectness	Imprecision	Publication bias	GRADE assessment	Explanation of GRADE assessment
**FACTORS RELATED TO DONATION DURATION**									
#1 Duration of donation	The duration of donation is positively associated with the volume of milk donated. The longer women donate their milk, the larger volume they donate.	Bocci et al., 2019 Quitadamo et al., 2018	Serious limitations. 1 study with low risk of bias (although unclear categories used as reference for regressions) and 1 study with high risk of bias (small sample size, unclear what percentage of the target population the 90 donors represent, no report of any regression analysis) **-1**	Inconsistency not explained **-1**	Not serious (evidence direct for comparison: population, phenomenon of interest, context)	Serious imprecisions: 2 studies, 1 with a small sample size. **-1**	Strongly suspected publication bias **-1**	Very low confidence ⨁○○○	One study with low concerns about methodological limitations and 1 study with very serious concerns. Unexplained inconsistency. Not serious indirectness (evidence direct for comparison: population, phenomenon of interest, setting), Serious imprecisions: 2 studies with a small sample size. Possible publication bias suspected.
#2 Start of donation	Women who started donating their milk sooner (before 4 months post-partum) donated larger volumes than women who started donated later (≥4 months post-partum).	Sierra-Colomina et al., 2014	Not serious. 1 study with low risk of bias (good methodology).	Only one study.	Not serious (evidence direct for comparison: population, phenomenon of interest, context)	Serious imprecision: Only one study. **-1**	Strongly suspected publication bias **-1**	Very low confidence ⨁○○○	One study with low risk of bias (good methodology). Only one study (unable to assess inconsistency). Not serious indirectness (evidence direct for comparison: population, phenomenon of interest, context), serious imprecision (only one study). Possible publication bias suspected.
**FACTORS RELATED TO INFANT HEALTH**									
#3 Preterm infants (gestational age)	Women who had a preterm infant donated larger volumes of human milk to milk banks compared to mothers of term infants.	Bocci et al., 2019 Jarmoc et al., 2021 Sierra-Colomina et al., 2014	Not serious. 3 studies with low risk of bias (good methodologies)	Inconsistency not explained **-1**	Not serious (evidence direct for comparison: population, phenomenon of interest, context)	Serious imprecision **-1**	Strongly suspected publication bias **-1**	Very low confidence ⨁○○○	Risk of bias not serious: 3 studies with low risk of bias (good methodology). Inconsistency not explained. Not serious indirectness. Serious imprecision between 3 studies. Possible publication bias suspected.
#4 Birth weight:	Infant birthweight correlated negatively with the volume of human milk donated by the mother. Mothers of infants with low birth weight donated more human milk.	Quitadamo et al., 2018	Very serious limitations. 1 study with high risk of bias (weak methodology: small sample size, unclear what percentage of the target population the 90 donors represent, no report of any regression analysis). **-2**	Only one study.	Not serious (evidence direct for comparison: population, phenomenon of interest, context)	Serious imprecisions: Only one study. **-1**	Strongly suspected publication bias **-1**	Very low confidence ⨁○○○	Very serious limitations regarding risk of bias (1 study with weak methodology: small sample size, unclear what percentage of the target population the 90 donors represent, no report of any regression analysis). Only one study (unable to assess inconsistency). Not serious indirectness Serious imprecisions (only one study). Possible publication bias suspected.

#5 Admission to the NICU	Two independent studies investigated the potential effect of admission of neonates to NICU on milk donation, finding diverging results. Meneses et al 2017 found that admission to NICU was associated with a lower prevalence of donation, while Nangia et al 2020 found that it was associated with larger volumes of human milk donation by mothers.	Meneses et al., 2017 Nangia et al., 2020	Serious limitations. 1 study with low risk of bias (good methodology) and 1 study with moderate risk of bias (no report of any regression analysis). **-1**	Inconsistency not explained **-1**	Not serious (evidence direct for comparison: population, phenomenon of interest, context)	Very serious imprecisions: Diverging effects found. **-2**	Strongly suspected publication bias **-1**	Very low confidence ⨁○○○	Serious limitations due to risk of bias: 1 study with low risk of bias (good methodology) and 1 study with moderate risk of bias (no report of any regression analysis). Inconsistency not explained. Not serious indirectness (evidence direct for comparison: population, phenomenon of interest, context). Very serious imprecisions: the two studies found diverging effects. Possible publication bias suspected.
#6 Thrush (in the infant)	Mothers who reported their infants had thrush donated more milk than mothers who reported their infant had not.	Osbaldiston & Mingle, 2007	Serious limitations. 1 study with moderate risk of bias (response rate < 35%, participants self-selected, small sample size. No report of any regression analysis). **-1**	Only one study.	Not serious indirectness (evidence direct for comparison: population, phenomenon of interest, context)	Serious imprecisions: Only one study. **-1**	Strongly suspected publication bias **-1**	Very low confidence ⨁○○○	Risk of bias: serious limitations, 1 study with moderate risk of bias (response rate < 35%, participants self-selected, small sample size. No report of any regression analysis). Not serious inconsistency (only one study). Not serious indirectness. Serious imprecisions: only one study. Possible publication bias suspected.
**FACTORS RELATED TO DONOR: Socio demographic characteristics**									
#7 Maternal age	Two independent studies investigated the potential influence of maternal age on the volume of human milk donation, finding diverging results. Maternal age was either positively (Quitadamo et al 2018) or negatively (Osbaldiston and Mingle 2007) associated with volume of milk donation.	Osbaldiston & Mingle, 2007 Quitadamo et al., 2018	Very serious limitations: 1 study with moderate risk of bias (response rate < 35%, participants self-selected, small sample size. No report of any regression analysis) and 1 study with high risk of bias (weak methodology: small sample size, unclear what percentage of the target population the 90 donors represent, no report of any regression analysis). **-2**	Inconsistency not explained -1	Not serious (evidence direct for comparison: population, phenomenon of interest, context)	Serious imprecision. 2 small sample size (<100) with diverging results found **-2**	Strongly suspected publication bias **-1**	Very low confidence ⨁○○○	Risk of bias: serious limitations, 1 study with moderate risk of bias (response rate < 35%, participants self-selected, small sample size) and 1 study with high risk of bias (weak methodology: small sample size, unclear what percentage of the target population the 90 donors represent, no report of any regression analysis). No report of any regression analysis). Inconsistency not explained .Not serious indirectness. Serious imprecision: 2 small sample size (<100 participants) with diverging results found. Possible publication bias suspected.
#8 Number of pregnancies	Women who have had 4 to 7 pregnancies have a higher likelihood of donation recurrence (donating their milk more than once) than women who have had 1 to 3 pregnancies.	Pimenteira Thomaz et al., 2008	Serious limitation: Moderate risk of bias (Independent and dependent variables were self-reported. Unclear how much the participation rate of eligible persons was). **-1**	Only one study.	Not serious (evidence direct for comparison: population, phenomenon of interest, context)	Serious imprecisions: Only one study. **-1**	Strongly suspected publication bias **-1**	Very low confidence ⨁○○○	Risk of bias: serious limitations, 1 study with moderate risk of bias (independent and dependent variables were self-reported. Unclear how much the participation rate of eligible persons was). Only one study (unable to assess inconsistency). Not serious indirectness (evidence direct for comparison: population, phenomenon of interest, context). Serious imprecisions (only one study). Possible publication bias suspected.

#9 Education level	Women with a higher education level have a higher likelihood of donation recurrence.	Pimenteira Thomaz et al., 2008	Serious limitation: Moderate risk of bias (Independent and dependent variables were self-reported. Unclear how much the participation rate of eligible persons was). **-1**	Only one study.	Not serious (evidence direct for comparison: population, phenomenon of interest, context)	Serious imprecisions: Only one study. **-1**	Strongly suspected publication bias **-1**	Very low confidence ⨁○○○	Risk of bias: serious limitations, 1 study with moderate risk of bias (independent and dependent variables were self-reported. Unclear how much the participation rate of eligible persons was). Only one study (unable to assess inconsistency). Not serious indirectness (evidence direct for comparison: population, phenomenon of interest, context). Serious imprecisions (only one study). Possible publication bias suspected.
#10 Profession	Women who were unemployed, homeworker, or workers donated significant smaller volume of milk than women with other professional categories.	Quitadamo et al., 2018	Very serious limitations. 1 study with high risk of bias (weak methodology: small sample size, unclear what percentage of the target population the 90 donors represent, no report of any regression analysis). **-2**	Only one study.	Not serious (evidence direct for comparison: population, phenomenon of interest, context)	Serious imprecisions: Only one study. **-1**	Strongly suspected publication bias **-1**	Very low confidence ⨁○○○	Very serious limitations regarding risk of bias (1 study with weak methodology: small sample size, unclear what percentage of the target population the 90 donors represent, no report of any regression analysis). Only one study (unable to assess inconsistency). Not serious indirectness Serious imprecisions (only one study). Possible publication bias suspected.
**FACTORS RELATED TO DONOR: Motivation to donate**									
#11 Excess of milk	When donors were asked about their motivations to donate, one of the variables was "Having an excess of milk" (on a 0-10 scale, "from not at all for this reason" to "very much for this reason"). A correlation was found between this variable and the volume of milk donated. Women who self-reported they donated because they had an excess of milk donated larger volume of milk than women who reported they did not donate their milk for this reason.	Osbaldiston & Mingle, 2007	Serious limitations. 1 study with moderate risk of bias (response rate < 35%, participants self-selected, small sample size. No report of any regression analysis). **-1**	Only one study.	Not serious (evidence direct for comparison: population, phenomenon of interest, context)	Serious imprecisions: Only one study. **-1**	Strongly suspected publication bias **-1**	Very low confidence ⨁○○○	Risk of bias: serious limitations, 1 study with moderate risk of bias (response rate < 35%, participants self-selected, small sample size. No report of any regression analysis). Only one study (unable to assess inconsistency). Not serious indirectness. Serious imprecisions: only one study. Possible publication bias suspected.
#12 Pumping to stimulate lactation	When donors were asked about their motivations to donate, one of the variables was "needed to pump to stimulate lactation" (on a 0-10 scale from "not at all for this reason" to "very much for this reason"). Donors who reported they donated because they needed to pump milk to stimulate lactation (answers 7-10 on the scale) donated larger volumes of milk than donors who reported they did not donate for this reason (answer 0 on the scale).	Osbaldiston & Mingle, 2007	Serious limitations. 1 study with moderate risk of bias (response rate < 35%, participants self-selected, small sample size. No report of any regression analysis). **-1**	Only one study.	Not serious (evidence direct for comparison: population, phenomenon of interest, context)	Serious imprecisions: Only one study. **-1**	Strongly suspected publication bias **-1**	Very low confidence ⨁○○○	Risk of bias: serious limitations, 1 study with moderate risk of bias (response rate < 35%, participants self-selected, small sample size. No report of any regression analysis). Only one study (unable to assess inconsistency). Not serious indirectness. Serious imprecisions: only one study. Possible publication bias suspected.
**FACTORS RELATED TO DONORS' FEATURES: Milk donation history**									

#13 Previous milk donation	Human milk donors who had already been milk donors previously donated significantly greater volumes of milk than women who had not previously donated.	Sierra-Colomina et al., 2014	Not serious. 1 study with low risk of bias (good methodology).	Only one study.	Not serious (evidence direct for comparison: population, phenomenon of interest, context)	Serious imprecisions: Only one study. **-1**	Strongly suspected publication bias **-1**	Very low confidence ⨁○○○	One study with low risk of bias (good methodology). Only one study (unable to assess inconsistency). Not serious indirectness (evidence direct for comparison : population, phenomenon of interest, context), serious imprecision (only one study). Possible publication bias suspected.

Supplementary TABLE S4b: CERQual Qualitative Evidence profile

	Summary of review finding	Studies contributing to the review	Methodological limitations	Coherence	Adequacy	Relevance[a]	CERQual assessment	Explanation of CERQual assessment
**FACTORS RELATED TO DONORS' INFANTS' FEATURES**								
#14 Baby feeding frequency	The frequency of baby's feeding was self-reported by some donors as having a potential positive and negative influence on the frequency of milk extraction and milk production.	Alencar & Seidl, 2010	Serious concerns about methodological limitations (1 study with serious concerns about methodological limitations [weak methodology]: small sample size and low participation rate).	No or very minor concerns about coherence	Serious concern about adequacy (only one study, offering thin data)	Moderate concern about relevance (partial relevance as the study is from two human milk banks in Brazil)	**Very low confidence** ⨁○○○	One study with serious concerns about methodological limitations. No or very minor concerns about coherence. Thin data from 1 country in South America, serious concerns regarding adequacy and moderate concerns regarding relevance.
#15 Growth of the baby	Baby's growth was self-reported by some donors as having a potential negative influence on the frequency of milk extraction and milk production.	Alencar & Seidl, 2010	Serious concerns about methodological limitations (1 study with serious concerns about methodological limitations [weak methodology]: small sample size and low participation rate).	No or very minor concerns about coherence	Serious concern about adequacy (only one study, offering thin data)	Moderate concern about relevance (partial relevance as the study is from two human milk banks in Brazil)	**Very low confidence** ⨁○○○	One study with serious concerns about methodological limitations. No or very minor concerns about coherence. Thin data from 1 country in South America, serious concerns regarding adequacy and moderate concerns regarding relevance.
**FACTORS RELATED TO DONORS' FEATURES: Health**								
#16 Self-hydration	Self hydration was self reported by donors as having a potential negative and/or positive influence on the frequency of milk extraction and milk production.	Alencar & Seidl, 2010	Serious concerns about methodological limitations (1 study with serious concerns about methodological limitations [weak methodology]: small sample size and low participation rate).	No or very minor concerns about coherence	Serious concern about adequacy (only one study, offering thin data)	Moderate concern about relevance (partial relevance as the study is from two human milk banks in Brazil)	**Very low confidence** ⨁○○○	One study with serious concerns about methodological limitations. No or very minor concerns about coherence. Thin data from 1 country in South America, serious concerns regarding adequacy and moderate concerns regarding relevance.
#17 Diet	Diet was self reported by donors as having a potential negative and/or positive influence on the frequency of milk extraction and milk production.	Alencar & Seidl, 2010	Serious concerns about methodological limitations (1 study with serious concerns about methodological limitations [weak methodology]: small sample size and low participation rate).	No or very minor concerns about coherence	Serious concern about adequacy (only one study, offering thin data)	Moderate concern about relevance (partial relevance as the study is from two human milk banks in Brazil)	**Very low confidence** ⨁○○○	One study with serious concerns about methodological limitations. No or very minor concerns about coherence. Thin data from 1 country in South America, serious concerns regarding adequacy and moderate concerns regarding relevance.

#18 Physical fatigue	Fatigue was self-reported by donors as having a potential negative influence on the frequency of milk extraction and milk production.	Alencar & Seidl, 2010	Serious concerns about methodological limitations (1 study with serious concerns about methodological limitations [weak methodology]: small sample size and low participation rate).	No or very minor concerns about coherence	Serious concern about adequacy (only one study, offering thin data)	Moderate concern about relevance (partial relevance as the study is from two human milk banks in Brazil)	**Very low confidence** ⊕○○○	One study with serious concerns about methodological limitations. No or very minor concerns about coherence. Thin data from 1 country in South America, serious concerns regarding adequacy and moderate concerns regarding relevance.
#19 Presence of negative emotions	Some donors self-reported the presence of negative emotions could have a negative influence on the frequency of milk extraction and milk production.	Alencar & Seidl, 2010	Serious concerns about methodological limitations (1 study with serious concerns about methodological limitations [weak methodology]: small sample size and low participation rate).	No or very minor concerns about coherence	Serious concern about adequacy (only one study, offering thin data)	Moderate concern about relevance (partial relevance as the study is from two human milk banks in Brazil)	**Very low confidence** ⊕○○○	One study with serious concerns about methodological limitations. No or very minor concerns about coherence. Thin data from 1 country in South America, serious concerns regarding adequacy and moderate concerns regarding relevance.

FACTORS RELATED TO DONORS' FEATURES: Motivation to donate

#20 Availability of time	Donors self-reported that availability of time they have (to pump) can have a negative or a positive influence on the frequency of milk extraction and milk production.	Alencar & Seidl, 2010	Serious concerns about methodological limitations (1 study with serious concerns about methodological limitations [weak methodology]: small sample size and low participation rate).	No or very minor concerns about coherence	Serious concern about adequacy (only one study, offering thin data)	Moderate concern about relevance (partial relevance as the study is from two human milk banks in Brazil)	**Very low confidence** ⊕○○○	One study with serious concerns about methodological limitations. No or very minor concerns about coherence. Thin data from 1 country in South America, serious concerns regarding adequacy and moderate concerns regarding relevance.

FACTORS RELATED TO DONORS' FEATURES: Breastfeeding and milk expression

#21 Experience of breastfeeding simultaneously	Milk donors reported a decreased in frequency donation as their milk production decreases by the process of breastfeeding itself (they do not have as much excess milk as they used to).	Machado et al., 2015	Minor concerns about methodological limitations (1 study with minor concerns about methodological limitations [good methodology]: unclear participants selection).	No or very minor concerns about coherence	Serious concern about adequacy (only one study, offering thin data)	Moderate concern about relevance (partial relevance as the study is from one setting in Spain)	**Low confidence** ⊕⊕○○	One study with minor concerns about methodological limitations. No or very minor concerns about coherence. Thin data from 1 country in Europe, serious concerns regarding adequacy and moderate concerns regarding relevance.
#22 Frequency of milk expression	Some donors self-reported that the frequency of milk expression has a potential positive influence on the frequency of milk extraction and milk production.	Alencar & Seidl, 2010	Serious concerns about methodological limitations (1 study with serious concerns about methodological limitations [weak methodology]: small sample size and low participation rate).	No or very minor concerns about coherence	Serious concern about adequacy (only one study, offering thin data)	Moderate concern about relevance (partial relevance as the study is from two human milk banks in Brazil)	**Very low confidence** ⊕○○○	One study with serious concerns about methodological limitations. No or very minor concerns about coherence. Thin data from 1 country in South America, serious concerns regarding adequacy and moderate concerns regarding relevance.

#23 Nothing interferes with milk production	Some donors self-reported that nothing interferes with milk production, which was seen as a positive influence on the frequency of milk extraction and milk production.	Alencar & Seidl, 2010	Serious concerns about methodological limitations (1 study with serious concerns about methodological limitations [weak methodology]: small sample size and low participation rate).	No or very minor concerns about coherence	Serious concern about adequacy (only one study, offering thin data)	Moderate concern about relevance (partial relevance as the study is from two human milk banks in Brazil)	**Very low confidence** ⨁○○○	One study with serious concerns about methodological limitations. No or very minor concerns about coherence. Thin data from 1 country in South America, serious concerns regarding adequacy and moderate concerns regarding relevance.
#24 Time of day for expressing milk	Donors self-reported that the period of the day might have a possible influence on the frequency of milk extraction and milk production.	Alencar & Seidl, 2010	Serious concerns about methodological limitations (1 study with serious concerns about methodological limitations [weak methodology]: small sample size and low participation rate).	No or very minor concerns about coherence	Serious concern about adequacy (only one study, offering thin data)	Moderate concern about relevance (partial relevance as the study is from two human milk banks in Brazil)	**Very low confidence** ⨁○○○	One study with serious concerns about methodological limitations. No or very minor concerns about coherence. Thin data from 1 country in South America, serious concerns regarding adequacy and moderate concerns regarding relevance.
**FACTORS RELATED TO DONORS: Other**								
#25 Mother's routines (going out, contraceptive use, return to work)	Some donors self-reported that going out, the use of contraception or them returning to work might have had a negative influence on the frequency of milk extraction and milk production.	Alencar & Seidl, 2010	Serious concerns about methodological limitations (1 study with serious concerns about methodological limitations [weak methodology]: small sample size and low participation rate).	No or very minor concerns about coherence	Serious concern about adequacy (only one study, offering thin data)	Moderate concern about relevance (partial relevance as the study is from two human milk banks in Brazil)	**Very low confidence** ⨁○○○	One study with serious concerns about methodological limitations. No or very minor concerns about coherence. Thin data from 1 country in South America, serious concerns regarding adequacy and moderate concerns regarding relevance.
**MILK BANK & HEALTH CARE RELATED FACTORS: Awareness and support**								
#26 Milk bank support to donation	The mother's environement (support offered by MB staff) has a positive influence on her willingness to continue donating her milk.	Machado et al., 2015	Minor concerns about methodological limitations (1 study with minor concerns about methodological limitations [good methodology]: unclear participants selection).	No or very minor concerns about coherence	Serious concern about adequacy (only one study, offering thin data)	Moderate concern about relevance (partial relevance as the study is from one setting in Spain)	**Low confidence** ⨁⨁○○	One study with minor concerns about methodological limitations. No or very minor concerns about coherence. Thin data from 1 country in Europe, serious concerns regarding adequacy and moderate concerns regarding relevance.
#27 Family support to donation	The support mothers reveive from their familiy has a positive influence on their willingness to continue donating their milk.	Machado et al., 2015	Minor concerns about methodological limitations (1 study with minor concerns about methodological limitations [good methodology]: unclear participants selection).	No or very minor concerns about coherence	Serious concern about adequacy (only one study, offering thin data)	Moderate concern about relevance (partial relevance as the study is from one setting in Spain)	**Low confidence** ⨁⨁○○	One study with minor concerns about methodological limitations. No or very minor concerns about coherence. Thin data from 1 country in Europe, serious concerns regarding adequacy and moderate concerns regarding relevance.

#28 Work impact and support	Milk donors reported that incomprehension and lack of support at their work place was an obstacle to remain a donor.	Machado et al., 2015	Minor concerns about methodological limitations (1 study with minor concerns about methodological limitations [good methodology]: unclear participants selection).	No or very minor concerns about coherence	Serious concern about adequacy (only one study, offering thin data)	Moderate concern about relevance (partial relevance as the study is from one setting in Spain)	**Low confidence** ⊕⊕○○	One study with minor concerns about methodological limitations. No or very minor concerns about coherence. Thin data from 1 country in Europe, serious concerns regarding adequacy and moderate concerns regarding relevance.
**MILK BANK & HEALTH CARE RELATED FACTORS: Logistics**								
#29 Distance to milk bank	Milk donors reported that the distance they have to travel to deliver their milk to the milk bank (no home collection service is available) is an obstacle to starting and remaining a milk donor.	Machado et al., 2015	Minor concerns about methodological limitations (1 study with minor concerns about methodological limitations [good methodology]: unclear participants selection).	No or very minor concerns about coherence	Serious concern about adequacy (only one study, offering thin data)	Moderate concern about relevance (partial relevance as the study is from one setting in Spain)	**Low confidence** ⊕⊕○○	One study with minor concerns about methodological limitations. No or very minor concerns about coherence. Thin data from 1 country in Europe, serious concerns regarding adequacy and moderate concerns regarding relevance.
#30 Human resources	Health care providers felt that shortage of human ressources in milk banks has a negative impact on the volume of milk collected.	Mondkar et al., 2018	Minor concerns about methodological limitation (1 study with minor concerns about methodological limitations [good methodology]: unclear recrutement process).	No or very minor concerns about coherence	Serious concern about adequacy (only one study, offering thin data)	Moderate concern about relevance (partial relevance as the study is from two health facilites in India)	**Low confidence** ⊕⊕○○	One study with minor concerns about methodological limitations. No or very minor concerns about coherence. Thin data from 1 country in Asia, serious concerns regarding adequacy and moderate concerns regarding relevance.

[a]To assess for indirect, partial or unclear relevance, consider: phenomenon of interest, population, setting, place, intervention, findings…

Grade certainty ratings:

Certainty	Meaning
Very low	The true effect is probably markedly different from the estimated effect
Low	The true effect might be markedly diferent from the estimated effect
Moderate	The authors believe that the true effect is probably close to the estimated effect
High	The authors have a lot of confidence that the true effect is similar to the estimated effect
